# Editorial: Physical activity in urban areas: Where and when?

**DOI:** 10.3389/fpubh.2026.1903268

**Published:** 2026-07-09

**Authors:** Hansen Li, Guodong Zhang, Yang Cao

**Affiliations:** 1 College of Physical Education, Sichuan Agricultural University, Yaan, China; 2 Institute of Sports Science, College of Physical Education, Southwest University, Chongqing, China; 3 Clinical Epidemiology and Biostatistics, School of Medical Sciences, Faculty of Medicine and Health, Örebro University, Örebro, Sweden; 4 Unit of Integrative Epidemiology, Institute of Environmental Medicine, Karolinska Institutet, Stockholm, Sweden

**Keywords:** active living, daytime, exercise, nighttime, physical activity, urban

## 1 Introduction

Physical activity can have numerous health benefits, whereas insufficient activity remains a global concern (1, 2). Recent studies have emphasized increasing activity through dose-related parameters, including duration, frequency, intensity, and total volume. However, these parameters do not fully capture the conditions under which activity occurs. The health implications of physical activity may depend on when and where it is performed. Exercising in the morning, evening, or late at night may involve different health-related consequences (3). Similarly, activity in polluted urban environments may not produce the same benefits as activity in cleaner and more supportive settings (4, 5).

Future research should therefore move beyond asking only whether people are active or how much activity they accumulate. Time and place should be treated as contextual factors that may shape, strengthen, weaken, or even reverse the relationship between physical activity and health. This approach can clarify where, when, for whom, and under what environmental conditions activity is most likely to be beneficial.

This Research Topic, *Physical Activity in Urban Areas: Where and When?*, was developed to advance understanding of the interaction between urban environments, temporal patterns, and physical activity-related health outcomes. The 13 articles included in this Research Topic show that physical activity is not only a matter of individual choice, but also a product of environmental affordances, perceived qualities, infrastructure, safety, accessibility, social rhythms, and policy decisions.

## 2 Overview of the Research Topic

The articles in this Research Topic cover diverse populations, settings, methods, and outcomes, including original studies, reviews, a brief research report, a mini review, and an opinion article. The Research Topic includes primarily studies from China and the Americas, including work on Hispanic families in the United States. A central contribution is that it links “where” and “when” as complementary dimensions of urban physical activity. Several studies focus on urban green spaces, parks, vegetation, perceived environmental quality, and restorative environments. Others examine university physical education, Open Streets programs, and culturally specific activity settings such as martial arts halls. Temporal issues are addressed through studies of park-use rhythms, university physical education as a structured time-space opportunity, and night running and cycling as nocturnal urban exercise.

## 3 Discussion of the contributing articles

One major theme is the role of urban green spaces as both physical settings and psychological resources for activity. Liu and Chao examined park-based physical activity in Zhengzhou using field observations, questionnaire data, and volunteered geographic information, showing that activity varied by time, location, demographic group, and perceived environmental conditions. Park activity clustered in specific zones and periods and was shaped by safety, facility convenience, environmental quality, noise, and crowding.

Several contributions extend this spatial perspective by examining psychological mechanisms. Zhang et al. found that perceived environmental quality in urban green spaces was positively associated with physical activity, with perceived restorativeness as a mediator and spatial accessibility as a moderator. Weng et al. used a photo-based experiment to show that higher vegetation levels increased physical activity intention largely through perceived environmental restorativeness. Lin et al. linked green space exposure with perceived stress, showing that mindfulness partly mediated this association and physical activity moderated key pathways. Together, these studies suggest that urban green spaces may promote activity not simply because they are available, but because they are perceived as safe, restorative, accessible, and inviting.

The health implications of green and outdoor activity were further developed in two reviews. Hu et al. synthesized randomized controlled trials on urban green exercise and mental health, providing evidence that green exercise may benefit mental health under certain intervention conditions. Wang et al. reviewed the relationship between green space and myopia among children and adolescents, broadening the health relevance of urban greenness beyond physical activity and highlighting benefits of outdoor exposure, visual environment, and opportunities for active living.

A second theme concerns the temporal organization of physical activity. Zhang et al. examined night running and night cycling, identifying drivers such as health awareness, changing work patterns, social media promotion, and the nighttime economy. Their review highlights potential benefits for cardiovascular health, stress reduction, and sleep, while emphasizing safety, air pollution, lighting, and infrastructure barriers. Liu and Chao also showed that park-based activity peaks during specific daily periods and differs between weekdays and weekends. Zhang et al. framed university physical education as a structured opportunity for health-oriented lifestyles, especially in later undergraduate years when participation may be voluntary. These studies demonstrate that the timing of physical activity is inseparable from work-rest cycles, institutional schedules, perceived safety, and daily routines.

A third theme concerns infrastructure, access, and equity. Gierbolini-Rivera et al. reviewed implementation strategies for Open Streets programs in the Americas, showing that temporary reallocation of streets from vehicles to people can promote physical activity and community engagement when supported by coalition building, local knowledge, needs assessment, and stakeholder involvement. Yao et al. investigated the spatial distribution of martial arts halls in China, revealing regional inequalities and showing how cultural heritage, population size, policy support, and education level shape activity spaces. Wei et al. found that walkability indicators such as intersection density and destination accessibility were associated with physical function in aging communities, with gender-specific patterns. Nissen et al. reported that neighborhood aesthetics and traffic safety were associated with selected physical activity outcomes in Hispanic children, although broader associations were modest. These studies show that activity-supportive environments must be interpreted through population needs, cultural context, age, gender, equity, and local infrastructure.

Khalil's opinion article offers a conceptual extension by linking urban physical activity infrastructure with neurogenesis and brain-derived neurotrophic factor. It suggests that walkable and cyclable environments may have implications for brain health, while warning that temperature extremes, air pollution, greenness, and tree placement may condition the benefits of urban physical activity.

Across the Research Topic, several advances are evident. The articles move beyond simple measures of physical activity volume and consider environmental perception, spatial accessibility, restoration, mindfulness, safety, facility distribution, and temporal patterns. They combine observational data, survey-based modeling, spatial analysis, experimental imagery, systematic review, scoping review, and conceptual synthesis. Together, they highlight that physical activity promotion in cities requires coordination between public health, urban planning, environmental design, education, transportation, and community governance.

Important gaps remain. Many empirical studies are cross-sectional, limiting causal inference. Several rely on self-reported physical activity or perceived environmental measures, while objective environmental exposure, device-measured activity, and longitudinal designs remain less common. Nighttime physical activity requires more field-based evidence on safety, lighting, air quality, thermal comfort, and health outcomes. Future studies should also address unequal access to safe and attractive activity spaces among children, older adults, women, low-income communities, and ethnic minority populations.

## 4 Conclusion

The contributions to this Research Topic demonstrate that promoting physical activity in urban areas requires more than encouraging individuals to move more. It requires designing and managing cities that make active living feasible across places, times, populations, and social contexts. Urban green spaces, walkable neighborhoods, safe streets, educational settings, culturally meaningful activity venues, and nighttime-friendly infrastructure all have roles to play, but their effectiveness depends on quality, accessibility, safety, environmental conditions, and daily routines. Taken together, this Research Topic advances a contextual understanding of urban physical activity. The question is not only how much physical activity people perform, but also where and when they perform it, why they choose particular spaces and times, and how urban environments can support healthier, more equitable, and more sustainable active lives.
